# Vascular Pattern Analysis for the Prediction of Clinical Behaviour in Pheochromocytomas and Paragangliomas

**DOI:** 10.1371/journal.pone.0121361

**Published:** 2015-03-20

**Authors:** Lindsey Oudijk, Francien van Nederveen, Cécile Badoual, Frédérique Tissier, Arthur S. Tischler, Marcel Smid, José Gaal, Charlotte Lepoutre-Lussey, Anne-Paule Gimenez-Roqueplo, Winand N.M. Dinjens, Esther Korpershoek, Ronald de Krijger, Judith Favier

**Affiliations:** 1 Department of Pathology, Erasmus MC Cancer Institute, Erasmus MC, University Medical Center, Rotterdam, the Netherlands; 2 Laboratory for Pathology, PAL Dordrecht, Dordrecht, the Netherlands; 3 Université Paris Descartes, Sorbonne Paris Cité, Faculté de Médecine, F-75006 Paris, France; 4 Assistance Publique-Hôpitaux de Paris (AP-HP), Hôpital Européen Georges Pompidou, Département d’anatomo-pathologie, F-75015 Paris, France; 5 Department of Pathology, Pitié-Salpetrière Hospital, AP-HP, Pierre and Marie Curie University, Sorbonne Universities, Paris, France; 6 INSERM U1016 CNRS UMR8104, Institut Cochin, Paris Descartes University, Sorbonne Paris Cité, France; 7 Department of Pathology, Tufts University School of Medicine & Tufts Medical Center, Boston, Massachusetts, United States of America; 8 Department of Medical Oncology, Erasmus MC Cancer Institute, Cancer Genomics Netherlands, Rotterdam, The Netherlands; 9 INSERM, UMR970, Paris Cardiovascular Research Center, F-75015 Paris, France; 10 Assistance Publique-Hôpitaux de Paris (AP-HP), Hôpital Européen Georges Pompidou, Service de Génétique, F-75015 Paris, France; 11 Department of Pathology, Reinier de Graaf Hospital, Delft, the Netherlands; Istituto dei tumori Fondazione Pascale, ITALY

## Abstract

Pheochromocytomas (PCCs) are neuroendocrine tumors arising from chromaffin cells of the adrenal medulla. Related tumors that arise from the paraganglia outside the adrenal medulla are called paragangliomas (PGLs). PCC/PGLs are usually benign, but approximately 17% of these tumors are malignant, as defined by the development of metastases. Currently, there are no generally accepted markers for identifying a primary PCC or PGL as malignant. In 2002, Favier et al. described the use of vascular architecture for the distinction between benign and malignant primary PCC/PGLs. The aim of this study was to validate the use of vascular pattern analysis as a test for malignancy in a large series of primary PCC/PGLs. Six independent observers scored a series of 184 genetically well-characterized PCCs and PGLs for the CD34 immunolabeled vascular pattern and these findings were correlated to the clinical outcome. Tumors were scored as malignant if an irregular vascular pattern was observed, including vascular arcs, parallels and networks, while tumors with a regular pattern of short straight capillaries were scored as benign. Mean sensitivity and specificity of vascular architecture, as a predictor of malignancy was 59.7% and 72.9%, respectively. There was significant agreement between the 6 observers (mean κ = 0.796). Mean sensitivity of vascular pattern analysis was higher in tumors >5 cm (63.2%) and in genotype cluster 2 tumors (100%). In conclusion, vascular pattern analysis cannot be used in a stand-alone manner as a prognostic tool for the distinction between benign and malignant PCC, but could be used as an indicator of malignancy and might be a useful tool in combination with other morphological characteristics.

## Introduction

Pheochromocytomas (PCCs) are neuroendocrine tumors derived from chromaffin cells of the adrenal medulla. Related tumors that arise from the paraganglia outside the adrenal medulla are called paragangliomas (PGLs). Paragangliomas can be further classified as parasympathetic or sympathetic, depending on their origin. Parasympathetic PGLs are mainly located in the head and neck and usually do not secrete catecholamines, unlike PCCs and sympathetic PGLs (located in the thorax, abdomen and pelvis) that usually produce adrenaline or noradrenaline.[1] About 40% of PCCs and PGLs carry a germline mutation in one of the following genes: *SDHA*, *SDHB*, *SDHC*, *SDHD* (referred to as *SDHx*), *RET*, *VHL*, *NF1*, *TMEM127*, *MAX*, *SDHAF2*, and the recently identified gene *FH (for review*, *see* [2]*)*. In addition, somatic mutations of *RET*, *VHL*, *NF1*, *MAX*, *HIF2A* and *HRAS* can be detected in a further 25–30% of these tumors.[3–6] *SDHx* and *VHL*-related PCC/PGL are referred to as ‘cluster 1’ tumors, following microarray gene expression studies, while ‘cluster 2’ includes the tumors with *RET*, *NF1*, *TMEM127*, and *MAX* mutations.[7,8]

Ten to 17% of PCCs and sympathetic PGLs are malignant, in which cases the prognosis of patients is poor and treatment basically palliative. Malignancy in PCC/PGL is defined by the 2004 World Health Organization classification as a chromaffin cell tumor with the presence of metastases to sites where chromaffin tissue should normally not be found (principally bones, liver, lymph nodes and lungs).[9,10] This definition distinguishes malignancy from multifocal disease. Local invasive growth of the tumor into other organs or major blood vessels, as has been reported to predict malignant behavior in other tumor types, does not fulfill the definition in PCC/PGL. The diagnosis of malignancy is clear-cut if metastatic lesions are present, but to date, no molecular or histologic markers exist to predict if a primary PCC/PGL has metastatic potential. Metastases can occur after a long latency period, sometimes more than 10 or even 20 years after diagnosis of the primary tumor.[11]

Currently, clinical risk factors for metastasis in patients with PCC/PGLs include tumor location, genotype, and size.[12] Patients with *SDHB*-related PCC/PGLs, extra-adrenal tumors or a primary tumor size over 5 cm have a higher risk to develop metastatic disease. In general, risk is highest for *SDHB*-mutated tumors (at least 30%), which are usually extra-adrenal and relatively large. Many studies have tried to find biomarkers to differentiate between benign and malignant PCC/PGL, such as the Ki67 labeling index, human telomerase reverse transcriptase (hTERT) or CD44 expression.[13,14] However, none of these markers allows a definite diagnosis of malignancy in PCC/PGL. Also, histologic criteria such as vascular invasion, mitotic activity, or cellular atypia cannot be used to definitely differentiate tumors with the potential to metastasize. The Pheochromocytoma of the Adrenal gland Scaled Score (PASS) was the first scoring system for the diagnosis of PCCs, which combined histopathological features to distinguish between benign and malignant tumors.[15] However, the reproducibility of the PASS has not been established.[16] Recently, Kimura et al. proposed the combination of GAPP (Grading of Adrenal Phaeochromocytoma and Paraganglioma) classification and SDHB immunohistochemistry for the prediction of metastasis in PCCs.[17]

PCCs and PGLs are highly vascularized tumors, even though there is a lot of variation in the vascular architecture of individual tumors. In 2002, we described the use of the vascular architecture for the distinction between benign and malignant PCC/PGLs.[18] We observed that malignant PCCs displayed a peculiar vascular pattern, which was not found in benign tumors. However, this published series was small and therefore the vascular pattern analysis was not implemented in routine PCC/PGL diagnostics. In the present study we determined, in a multi-center international collaboration, the vascular pattern of 184 genetically well-characterized PCCs and PGLs. The results of these analyses were correlated to the clinical behavior of the tumors. This allows determining the role of vascular pattern analysis in the initial pathological work-up of PCC and PGL patients.

## Materials and Methods

We analyzed 88 paraffin-embedded tumors collected from 72 PCC/PGL patients collected from the archives of the Department of Pathology of the Erasmus MC Cancer Institute, Erasmus MC, University Medical Center (Rotterdam, the Netherlands) and 96 tumors from 87 patients collected from Paris by the French COMETE network from patients operated in two referral centers in Paris (Hôpital Européen Georges Pompidou and Hôpital Cochin).

The series comprised 59 malignant tumors and 125 benign samples. As defined by the World Health Organization (WHO) in 2004 [19], and by the international guidelines of the Endocrine Society [20] diagnosis of malignancy was based on the presence of a metastasis at the site were chromaffin cells are usually absent. A summary of the clinicopathological characteristics of the tumors is provided in Table 1. The Dutch tissues were used in accordance with the code of conduct Proper Secondary Use of Human Tissue established by the Dutch Federation of Medical Scientific Societies (http://www.federa.org). The study obtained the institutional review board approval [Medical Ethical Committee from Erasmus MC University Medical Center, Rotterdam, The Netherlands] and patients gave their informed written consent. For French tissues, informed signed consent was obtained from each patient recruited by the COMETE network, and the study was formally approved by an institutional review board [Comité de Protection des Personnes (CPP) Ile de France III, June 2012].

**Table 1:** Clinicopathological characteristics.

*Mutated gene*	*No*. *of tumors*	*Sex M/F*	*Age range (yr; mean)*	*PCC*	*PGL*	*Meta*	*B/M*	*Tumor size (mm; mean)*	*FU (yr; mean)*
***VHL* germline**	20	13/7	7–50 (22.3)	16	4	0	17/3	25–80 (47.8)	9.2
***VHL* somatic**	7	2/5	17–56 (37.1)	5	2	0	4/3	18–110 (62)	10.83 (N = 1D)
***RET* germline**	24	4/20	16–76 (38.3)	24	0	0	24/0	9–110 (32.3)	9.97 (N = 1D)
***RET* somatic**	2	1/1	49–62 (55.5)	2	0	0	2/0	NA	7.5
***NF1* germline**	13	4/8 (1U)	26–65 (40)	13	0	0	13/0	25–70 (48.3)	7.75
***NF1* somatic**	5	4/1	37–73 (49.6)	4	0	1	3/2	60–80 (38.3)	7.6
***SDHA***	2	1/1	32	1	1	0	2/0	60–72 (66)	0.5
***SDHB***	20	9/11	10–63 (36.6)	8	10	2	5/14 (1U)	40–140 (90.9)	9.83 (N = 4D)
***SDHC***	1	0/1	16	0	1	0	1/0	45	NA
***SDHD***	8	8/0	16–62 (36)	2	6	0	4/4	20–100 (51.3)	15.83
***TMEM127***	2	0/2	44	2	0	0	2/0	NA	15
***HRAS* somatic**	1	0/1	61	1	0	0	1/0	NA	9 (D)
***FH***	1	0/1	63	0	0	1	0/1	40	10.5 (D)
**SPOR**	78	37/41	9–79 (48.6)	61 (2U)	10	5	46/32	7–130 (60.5)	7.20 (N = 1D)

M, male; F, female; U, unknown; PCC, pheochromocytoma; PGL, paraganglioma; Meta, metastasis; B, benign; M, malignant; FU, follow-up; D, died.

Immunohistochemical analysis was performed by staining endothelial cells with CD34 monoclonal antibody on 4–6 μm sections of formalin-fixed paraffin-embedded tumors.

For French slides, the anti-CD34 (IM0786, Beckman Coulter) was used at a 1:500 dilution. After deparaffinization, rehydration and H2O2 treatment, tissues were blocked in goat serum for 30 minutes and the primary antibody was applied at room temperature for 1h. The biotinylated secondary anti-rabbit antibody (Vector Lab; 1:400) was applied for 45 minutes and the revelation was assessed using histogreen kit (Vector Laboratories, EUROBIO/ABCYS, Les Ulis, France). Slides were counterstained with Nuclear Fast red and coverslipped.

For Dutch samples, a 1:75 dilution of the CD34 monoclonal antibody (clone QBEnd/10; Neomarkers) was used, together with a protocol based on the Ventana BenchMark Ultra System (Ventana Medical Systems, Inc, Tucson, AZ). After deparaffinization, slides were submitted to heat-induced epitope retrieval in Ventana Cell Conditioning 1 (pH 8.4) at 99°C for 64 minutes. Endogenous peroxidase was blocked using 3% hydrogen peroxide. The diluted CD34 antibody was applied and incubated for 32 minutes at 36°C. The stains were developed using 3,3'-diaminobenzidine (DAB) as chromogene. Finally, the slides were counterstained in haematoxylin, washed in water, dehydrated through graded alcohol and coverslipped.

In all cases, negative control experiments were performed by omitting the primary antibody.

Based on the results from the study by Favier et al., a tutorial (training set) with representative immunohistochemical images of vascular patterns related to benign and malignant tumors (see S1–S4 Figs.) was distributed in advance among the 6 observers (1 research scientist (JF) and 5 trained pathologists (CB, RK, FN, FT, AT)). After this, the immunohistochemical images of the 184 tumors were independently evaluated by the 6 observers. All tumors had to be scored into 4 groups according to their vascular architecture: benign (B+), probably benign (B-), probably malignant (M-) and malignant (M+).

### Statistical analysis

To calculate the interobserver agreement the Kappa test was performed. The Chi-square test was used to associate the prediction of the observers with clinicopathological characteristics of the samples, i.e. clinical behavior, tumor size, genotype. Two-sided P values <0.05 were considered as statistically significant. Statistical tests were performed using Analyse-it v2.26 (Analyse-it Software, Ltd. Leeds, United Kingdom).

## Results

**Fig 1:**
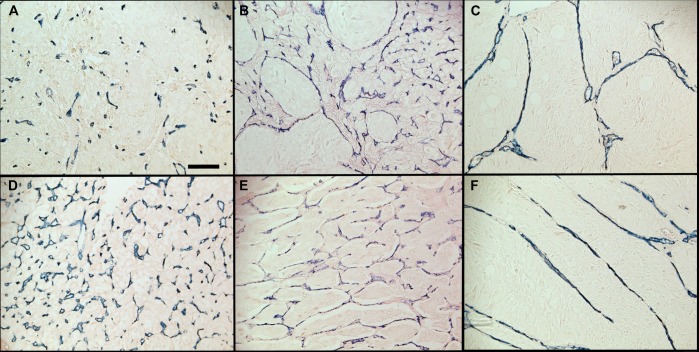
Vascular architecture in PCC/PGLs. Immunostaining of blood vessels with anti-CD34 reveals a homogenously distributed vascular pattern in benign tumors (A, D), while malignant tumors display irregularity (B) and vascular structures forming arcs (C), networks (E) and parallels (F). All panels are at the same magnification. Scale bar = 100μm.

The vascular architecture of 184 PCC/PGL tumors was revealed by labeling endothelial cells using CD34 immunohistochemistry. Tumors were blindly scored as “probably benign” (B-), “certainly benign” (B+), “probably malignant” (M-), or “certainly malignant” (M+) according to their vascular pattern by six expert pathologists. If the pattern was distributed regularly throughout the whole tissue section, consisting mostly of short, straight vascular segments, tumors were designated as benign. In contrast, if a discontinuous distribution of blood vessels (i.e. highly vascularized zones adjacent to avascular areas) and vascular structures forming arcs, parallels and networks could be identified, tumors were scored as malignant (Fig. 1 and S2–S4 Figs.).

### The interobserver agreement (Kappa agreement)

The interobserver agreements between all 6 observers after sorting the tumors into 4 groups (B+, B-, M-, M+) ranged from a fair to moderate agreement (Table 2). The highest agreement was reached between observer 2 and 3 (κ = 0.596) and the lowest between observer 2 and 5 (κ = 0.351).

**Table 2:** Interobserver agreement (Kappa test) of benign, probably benign, probably malignant and malignant tumors (4 groups).

	*Observer 1*	*Observer 2*	*Observer 3*	*Observer 4*	*Observer 5*
Observer 2	0.546				
Observer 3	0.570	0.596			
Observer 4	0.554	0.525	0.552		
Observer 5	0.424	0.351	0.432	0.471	
Observer 6	0.429	0.393	0.459	0.397	0.430

All agreements p<0.0001.

When the tumors were sorted into 2 groups (B+/- vs M+/-) the Kappa agreement was substantial to almost perfect (Table 3). Observer 1 and 4 displayed the highest agreement (κ = 0.897) and observer 2 and 5 the lowest ((κ = 0.725).

**Table 3:** Interobserver agreement (Kappa test) of (probably) benign versus (probably) malignant tumors (2 groups).

	*Observer 1*	*Observer 2*	*Observer 3*	*Observer 4*	*Observer 5*
Observer 2	0.852				
Observer 3	0.849	0.842			
Observer 4	0.897	0.803	0.831		
Observer 5	0.779	0.725	0.747	0.785	
Observer 6	0.783	0.765	0.773	0.788	0.727

All agreements p<0.0001.

### Vascular architecture as a prognostic tool

For each observer, the association between the predicted clinical outcome with the true benign or malignant status of the tumor was investigated. The specificity of this approach, defined as the percentage of benign cases correctly predicted as benign, ranged from 55.3%–79.8% (mean 72.9%). The sensitivity, defined as the percentage of malignant cases correctly predicted as malignant, ranged from 49.2%–71.9% (mean 59.7%). Observer 1 was the best predictor, with a Youden index of 0.493 (Table 4).

Overall, 35 of 59 (sensitivity is 59%) malignant PCCs were scored as malignant by the majority (i.e. ≥4 of 6) of the observers. For benign tumors, there were 94 of 124 (specificity is 76%) tumors that were correctly predicted to be benign. In 13 benign tumors all observers scored the tumor as malignant.

**Table 4:** Associating predicted benign/malignant call with TRUE benign/malignant status.

	Observer 1	Observer 2	Observer 3	Observer 4	Observer 5	Observer 6
Sensitivity	69.5%	50.8%	50.9%	66.1%	71.9%	49.2%
Specificity	79.8%	74.0%	74.1%	77.4%	55.3%	76.6%
PPV	62.1%	48.4%	48.3%	58.2%	44.6%	50.0%
NPV	84.6%	75.8%	76.1%	82.8%	79.7%	76.0%
Pval	8.3E-11	0.0009	0.0012	1.1E-08	0.0008	0.0005
Youden	0.493	0.248	0.250	0.435	0.272	0.258

Sensitivity is defined as the percentage of TRUE malignant cases correctly predicted as malignant

Specificity is defined as the percentage of TRUE benign cases correctly predicted as benign

Pval: p-value Chi-square test

PPV: positive predictive value

NPV: negative predictive value

Youden is defined as sensitivity+specificity-1. The higher the Youden, the better the prediction.

Taking tumor size into consideration, malignant tumors were significantly more often greater than 5 cm compared to benign tumors (P = 0.0233). Mean sensitivity of vascular architecture as a prognostic tool in tumors >5 cm was 63.2% (range 50.0%–78.3%), while in smaller tumors (<5 cm) the mean sensitivity was 57.5% (range 30.58%–76.8%). Mean specificity was 66.3% (range 50.0%–73.5%) and 80.8% (range 71.7%–85.7%) in tumors >5 cm and <5 cm, respectively.

After splitting up the tumors according to genotype cluster (i.e. cluster 1: *SDHx*- and *VHL*-related PCC/PGL; cluster 2: *RET*-, *NF1*-, and *TMEM127*-related tumors), malignant tumors (n = 24) clustered more often in genotype cluster 1 (P<0.0001), compared to benign tumors (n = 33). Mean sensitivity of vascular pattern analysis in genotype cluster 1 tumors was 52.3% (range 33.3%–68.2%) and the mean specificity 69.1% (range 57.1%–78.8%). Mean sensitivity in cluster 2 tumors (n = 52, of which 49 benign and 3 malignant) was 100% (range 100%–100%) and the mean specificity 77.4% (range 57.58%–57.8%).

## Discussion

Currently it is still not possible to state with certainty whether a primary PCC or PGL is benign or malignant. The diagnosis of malignancy depends on the occurrence of metastases, which is rare and can have a latency of many years.[11] Considering the concept that induction of angiogenesis is necessary for tumor growth and metastases, and because PCCs are highly vascularized tumors, we analyzed the vascular pattern of 184 PCC/PGL, immunohistochemically stained with anti-CD34 antibody. Mean sensitivity and specificity of vascular architecture, as a predictor of malignancy was 59.7% and 72.9%, respectively. There was significant agreement between the 6 observers (mean κ = 0.796). Mean sensitivity of vascular pattern analysis was higher in tumors >5 cm (63.2%) and in genotype cluster 2 tumors (100%).

The rationale of this study is based on a previous study where we observed an abnormal and discontinuous vascular pattern, including vascular arcs, parallels and networks in 9 of 9 malignant PCCs[18]. On the contrary, 9 of 10 benign tumors in that series showed a homogenous pattern of short straight capillaries. The biological significance of these differences in vascular pattern between benign and metastatic PCC/PGL is unclear. Whether the irregularity in the vascular architecture of malignant tumors corresponds to different steps in the angiogenic process or to a specific growth pattern of tumor cells is difficult to apprehend in the context of the actual study. It may anyhow have an influence on the oxygenation status of tumor cells and may reflect a capacity of malignant cells to resist to hypoxia. Since the initial cohort of 19 tumors was small (and of incomplete genotyping, at that time), we validated vascular pattern analysis in a larger cohort, fully characterized at the molecular level, and with multiple observers. In the current study, 35 of 59 (59%) malignant PCCs were scored as malignant by a majority of the observers. In contrast, 94 of 124 (76%) benign tumors were correctly predicted to be benign. We could not confirm the high sensitivity of 100% using vascular pattern analysis for the distinction between benign and malignant PCC, as reported in the initial study.

The Pheochromocytoma of the Adrenal gland Scaled score (PASS) was developed in 2002 by Thompson, to distinguish benign from malignant PCC by histopathological parameters.[15] The following features (with values) were scored: large cell nests or diffuse growth (2), central or confluent tumor necrosis (2), high cellularity (2), cellular monotony (2), tumor cell spindling (2), mitotic figures more than 3 per 10 HPF (2), atypical mitotic figures (2), extension into adipose tissue (2), vascular invasion (1), capsular invasion (1), profound nuclear pleomorphism (1) and nuclear hyperchromasia (1). Each histopathological feature of the PASS score was given a value of 1 or 2 and these values are summed up. Of the 50 metastatic tumors investigated in that study, all had a PASS score of more than 4. However, the study also reported that 17 of the 50 benign tumors had a score of more than 4 as well (with a follow-up period of 5 years).[15] Hence, an inappropriately high proportion of tumors were suggested to have malignant potential. This overestimation of malignant tumors is something we also encountered in the present study, although our specificity was higher (76% versus 66%).

Later, in a study by Wu et al. in 2009, five endocrine pathologists investigated the applicability of the PASS and it showed a large inter- and intra-observer variation [16]. In contrast, we found a substantial interobserver agreement (mean κ = 0.796) among 6 observers recognizing vascular patterns, indicating that the vascular pattern classification system used in our study is a much less complicated tool. However, because of low sensitivity and specificity the system is unlikely to be useful for stratifying risk of metastasis. Despite this disappointing outcome, it is of interest that several of the vascular patterns might actually provide means to enhance recognition of histologic parameters scored in the PASS including diffuse growth (Fig. 1C and 1F) and large cell nests (Fig. 1B). The latter are also a component of the risk stratification system proposed by Kimura et al.[17]

Many studies have reported the evaluation of angiogenesis in PCC/PGL by examining the microvascular density (MVD). Most of these studies suggest an increase in MVD in malignant versus benign tumors.[21–24] However, the quantification of MVD appeared not to be a reliable predictor of malignancy, which is confirmed by other studies reporting the absence of a statistical association between microvascular counts and malignancy.[25] MVD counting can be based on the number, length or branching of the vessels, which will definitely influence the results. To our knowledge, our initial study [18] and the present study are the only ones focusing on the difference in vascular architecture between benign and malignant PCC/PGL, instead of blood vessel counting.

Although the immunohistochemical stainings were performed in two different medical university centers, there was no bias due to the origin of the section, which was also demonstrated by the kappa test.

Twenty-four clinically benign tumors (corresponding to 23 patients) in the present series were scored as malignant by ≥4 observers (S1 Table). Metastasis can occur up to 20 or even 30 years after the occurrence of a primary pheochromocytoma. We have addressed whether the follow-up was sufficient to clearly establish the benign status of these tumors. For eight tumors (7 patients), we had no follow-up data (most corresponded to surgeries performed at least 25 years ago). One patient has died due to unrelated disease, and one (scored as malignant by 5 observers) actually became metastatic recently. For the 15 other patients, median follow-up was of 10 ± 6.4 years (range 2.3–19). Hence, although the follow-up is quite important for these patients, we cannot exclude that some of these patients will develop a metastatic form of their disease in the future.

In particular, 13 benign PCCs of 12 patients were scored as malignant by all 6 observers, so attention should be paid to the follow-up of these patients. The corresponding cases included 4 *RET* (2 germline and 1 somatic mutation), 2 *VHL* (1 germline and 1 somatic mutation) and 6 sporadic PCCs.

In summary, we tested vascular architecture as a prognostic tool in 184 PCC/PGL after CD34 immunolabeling. Tumors were scored as malignant if a discontinuous vascular pattern was observed, including vascular arcs, parallels and networks, while tumors with a uniform pattern of short straight capillaries were scored as benign. There was significant agreement between observers, indicating that there is a real variance between the different vascular patterns. However, the mean sensitivity, defined as the percentage of TRUE malignant cases correctly predicted as malignant, was only 59.7%. Accordingly, vascular pattern analysis cannot be used in a stand-alone manner as a prognostic tool for the distinction between benign and malignant PCCs. However, understanding the mechanisms responsible for the development of different patterns might provide new information on tumor biology and ultimately prove to have clinical value.

## Supporting Information

S1 Fig Vascular architecture of the adrenal medulla. Pictures are shown at a 10X (A) and a 20X (B) magnification. Scale bars = 100μm.
(TIF)

S2 Fig Vascular architecture in benign tumors. Typical vascular architecture of benign PCC/PGLs are shown for a cluster 2 patient (A, B) and a cluster 1 patient (C, D). Pictures were taken at a 10X (A, C) and a 20X (B, D) magnification. Scale bars = 100μm.
(TIF)

S3 Fig Vascular architecture in malignant tumors. Typical vascular patterns seen in malignant PCC/PGLs are shown for 5 different tumors. These examples illustrate the irregular pattern (A, B), the presence of networks (C-F), arcs (G, H) and parallels (I, J). Pictures are shown at a 10X (A, C, E, G, I) and a 20X (B, D, F, H, J) magnification. Scale bars = 100μm.
(TIF)

S4 Fig Typical example of classification. Patients were sorted in a blinded manner into 4 groups regarding to their vascular architecture revealed by CD34 immunostaining of endothelial cells and considered as certainly benign (A), probably benign (B), probably malignant (C) and certainly malignant (D). Scale bars = 100μm
(TIF)

S1 Table Apparently benign tumors classified as malignant according to the vascular pattern. Score attributed by each of the 6 observers: benign (B+), probably benign (B-), probably malignant (M-) and malignant (M+). F-U (Follow-up).
(DOCX)
