# Supplementary figures and images for: Vascular Pattern Analysis for the Prediction of Clinical Behaviour in Pheochromocytomas and Paragangliomas
Source: PLoS One. 2015 Mar 20;10(3):e0121361. doi: 10.1371/journal.pone.0121361 (PMC4368716; doi:10.1371/journal.pone.0121361)

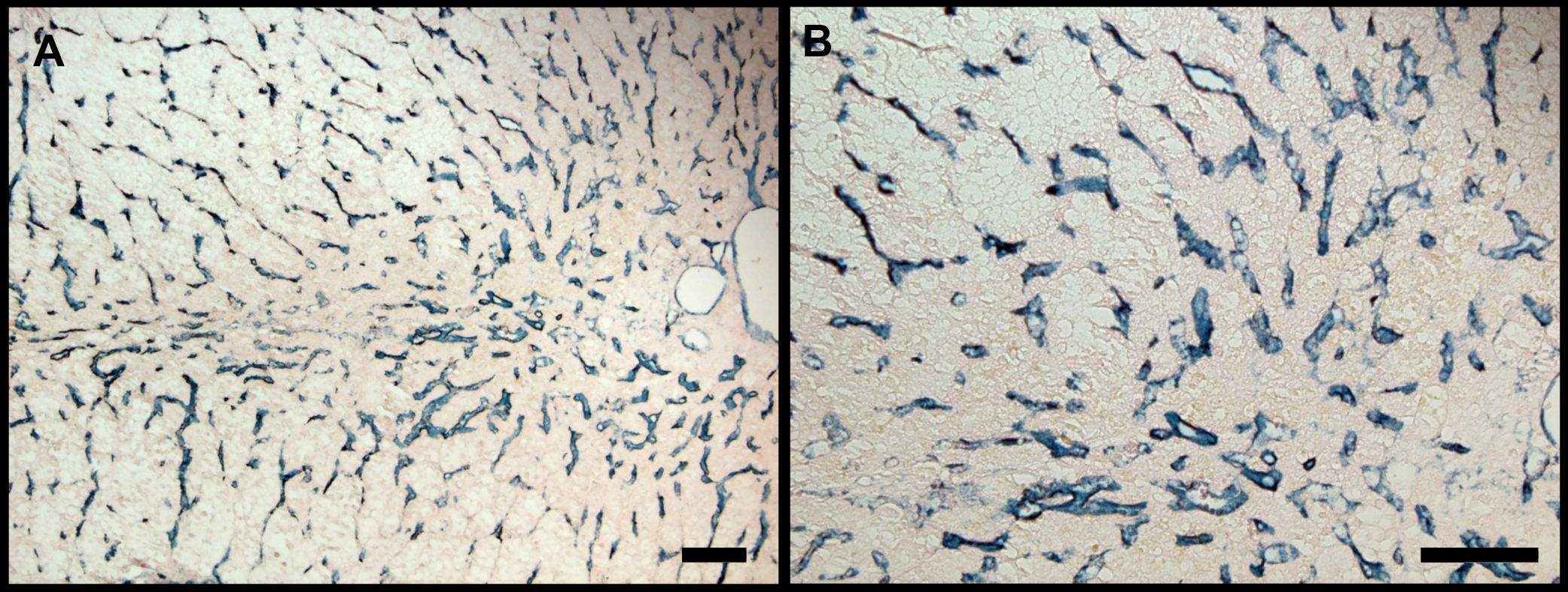

Supplement: S1 Fig — Pictures are shown at a 10X (A) and a 20X (B) magnification. Scale bars = 100μm. (TIF) [file pone.0121361.s001.tif]

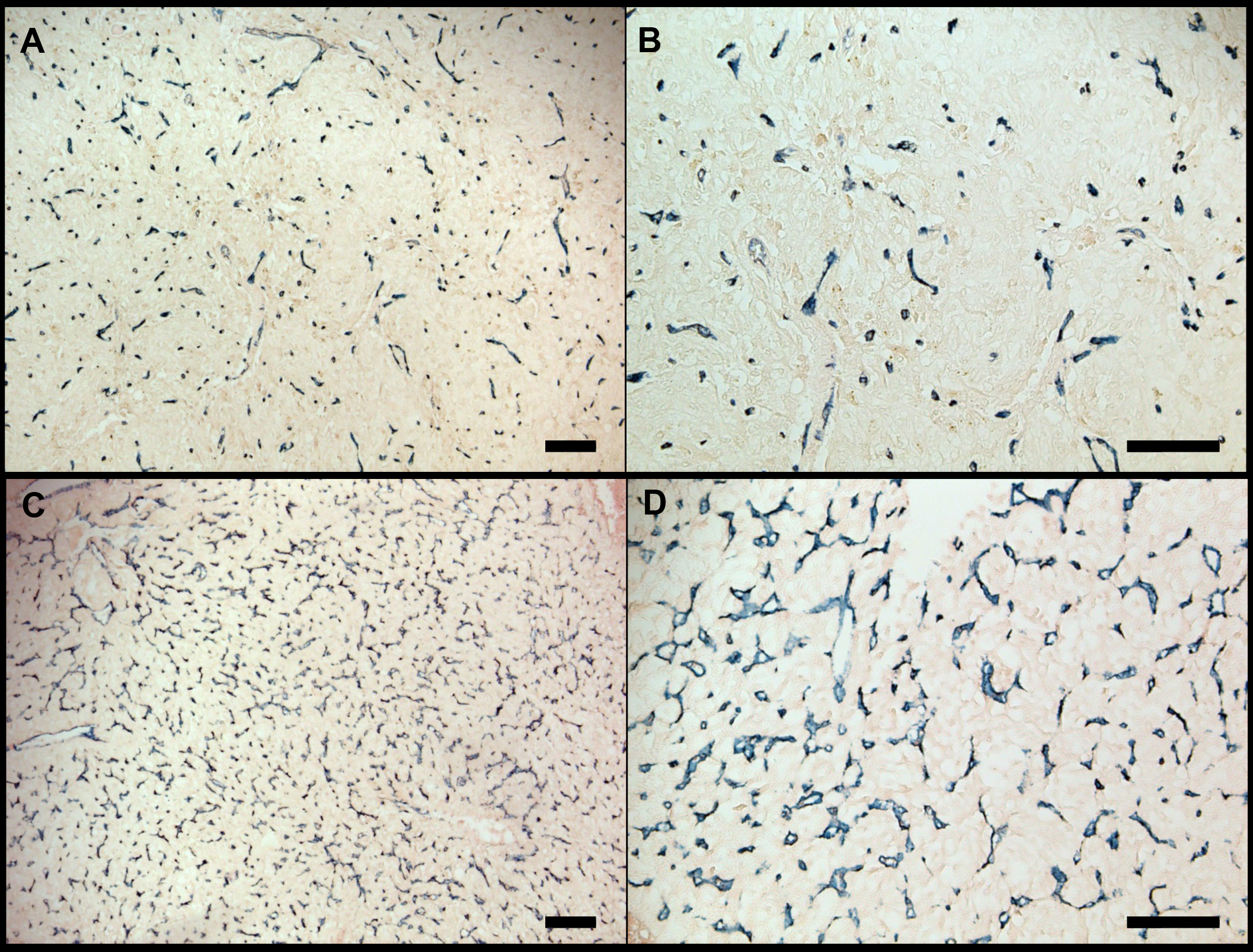

Supplement: S2 Fig — Typical vascular architecture of benign PCC/PGLs are shown for a cluster 2 patient (A, B) and a cluster 1 patient (C, D). Pictures were taken at a 10X (A, C) and a 20X (B, D) magnification. Scale bars = 100μm. (TIF) [file pone.0121361.s002.tif]

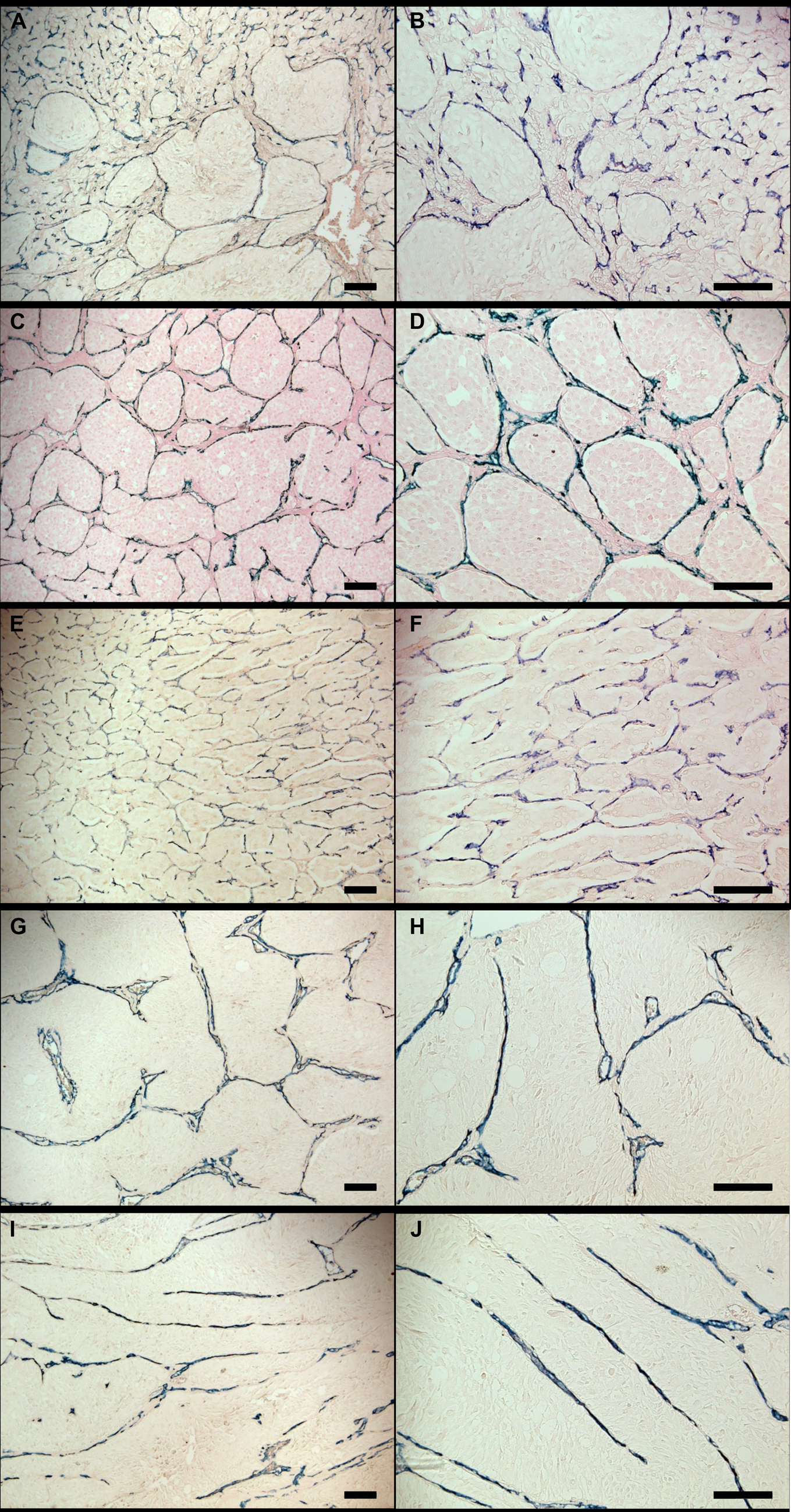

Supplement: S3 Fig — Typical vascular patterns seen in malignant PCC/PGLs are shown for 5 different tumors. These examples illustrate the irregular pattern (A, B), the presence of networks (C-F), arcs (G, H) and parallels (I, J). Pictures are shown at a 10X (A, C, E, G, I) and a 20X (B, D, F, H, J) magnification. Scale bars = 100μm. (TIF) [file pone.0121361.s003.tif]

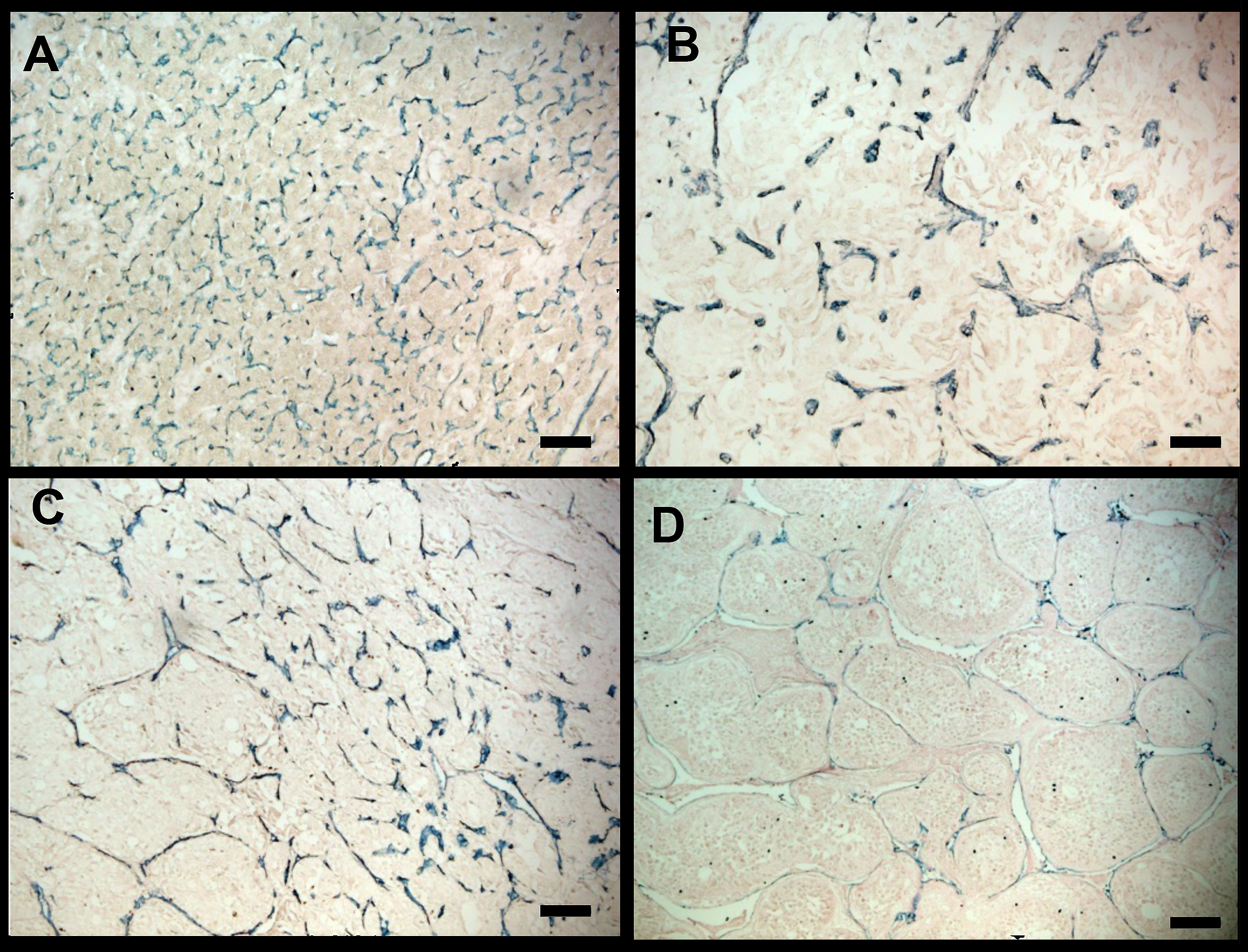

Supplement: S4 Fig — Patients were sorted in a blinded manner into 4 groups regarding to their vascular architecture revealed by CD34 immunostaining of endothelial cells and considered as certainly benign (A), probably benign (B), probably malignant (C) and certainly malignant (D). Scale bars = 100μm (TIF) [file pone.0121361.s004.tif]
