# Supplementary material for: Vascular Pattern Analysis for the Prediction of Clinical Behaviour in Pheochromocytomas and Paragangliomas
Source: PLoS One. 2015 Mar 20;10(3):e0121361. doi: 10.1371/journal.pone.0121361 (PMC4368716; doi:10.1371/journal.pone.0121361)
Supplement: S1 Table — Score attributed by each of the 6 observers: benign (B+), probably benign (B-), probably malignant (M-) and malignant (M+). F-U (Follow-up). (DOCX) [file pone.0121361.s005.docx]

**Supplementary Table 1 – Apparently benign tumors classified as malignant according to the vascular pattern.**  Score attributed by each of the 6 observers: benign (B+), probably benign (B-), probably malignant (M-) and malignant (M+). F-U (Follow-up)

| **Number** | **Observer1** | **Observer2** | **Observer3** | **Observer4** | **Observer5** | **Observer6** | **Gene mutated** | **Follow-up (months)** | **Current status** |
| --- | --- | --- | --- | --- | --- | --- | --- | --- | --- |
| 1 | M- | M- | M- | M- | - | B- | NF1 | 27,2 |  |
| 2 | M- | M+ | M+ | M+ | M+ | M- | RET | 33,3 |  |
| 3 | M- | M+ | M+ | B- | M+ | B+ | spor | 46,1 |  |
| 4 | M+ | M- | M+ | M+ | M+ | M- | spor | 58,3 |  |
| 5 | M- | M+ | M- | M- | M- | M- | RETs | 59,5 |  |
| 6 | M- | M- | M- | M- | M- | M- | spor | 67,0 | deceased |
| 7 | M+ | M+ | M- | M+ | M- | M- | spor | 92,8 |  |
| 8 | M+ | M- | M+ | M+ | M+ | M- | VHL | 108,3 |  |
| 9 | M- | M+ | M- | M- | M- | B- | VHL | 119,7 |  |
| 10 | M- | M+ | M- | M- | M- | M- | spor | 145,3 |  |
| 11 | B- | M- | B- | M+ | M+ | M- | SDHD | 158,5 |  |
| 12 | M- | M- | M+ | M+ | M+ | M- | spor | 178,8 |  |
| 13 | M- | M- | M- | M- | B- | B- | VHL | 199,7 |  |
| 14 | M- | B+ | B+ | M- | M- | M- | SDHB | 226,3 |  |
| 15 | M+ | M- | M+ | M- | M- | B- | SDHA | 283,0 |  |
| 16 | M+ | M+ | M+ | M+ | M+ | M- | VHLs | No F-U |  |
| 17 | M+ |  | B+ | M+ | M- | M- | spor | No F-U |  |
| 18 | M+ | M+ | M- | M+ | M- | M- | none | No F-U |  |
| 19 | B- | M- | M- | B- | M+ | M+ | spor | No F-U |  |
| 20 | M+ | M- | M+ | M+ | M+ | M- | RETs | No F-U |  |
| 21 | M- | B+ | M+ | M- | M+ | M- | spor | No F-U |  |
| 22 | M+ | M+ | M+ | M+ | M+ | M- | RETs | No F-U | deceased |
| 23 | M+ | M+ | M+ | M+ | M- | M+ | spor | No F-U |  |
| 24 | M- | M- | M+ | M+ | M- | B- | SDHB | 84,3 | malignant |
|  |  |  |  |  |  |  |  |  |  |
